# Phosphate solubilizing bacteria from soils with varying environmental conditions: Occurrence and function

**DOI:** 10.1371/journal.pone.0289127

**Published:** 2023-12-08

**Authors:** Walid Janati, Rachid Bouabid, Karima Mikou, Lahsen El Ghadraoui, Faouzi Errachidi

**Affiliations:** 1 Department of Biology, Functional Ecology and Environment Engineering Laboratory, Faculty of Science and Technology, Sidi Mohamed Ben Abdellah University, Fez, Morocco; 2 Department of Agronomy, National School of Agriculture, Meknes, Morocco; National Institute of Agricultural Research - INRA, MOROCCO

## Abstract

Phosphate solubilizing bacteria (PSB) is an advantageous way to supply phosphate (P) to plants. The Mediterranean climate of Morocco, especially the low-lying areas, is semi-arid with nutrient-depleted soils in which small-scale, low-income farmers dominate without access to expensive inorganic fertilizers. However, there is not a wide range of PSBs suitable for various agroecological situations. Furthermore, our understanding of the soil and climatic variables that influence their development is limited. This study aims to examine the impacts of specific environmental factors, such as climate and soil, on the abundance, potential, and diversity of PSBs in four agricultural regions of Morocco. To assess the possible impact of these factors on the P solubilization capacity of PSBs and plant growth-promoting (PGP) traits, we analyzed the soil and climate of each sample studied. Similarly, we tested the P solubilization efficiency of the isolates. The bacteria were isolated in a National Botanical Research Institute’s phosphate (NBRIP) agar medium. A total of 51 PSBs were studied in this work. The P-solubilization average of Rock P (RP) and Tricalcium P (TCP) of all strains that were isolated from each of the four regions ranged from 18.69 mg.L^-1^ to 40.43 mg.L^-1^ and from 71.71 mg.L^-1^ to 94.54 mg.L^-1^, respectively. The PGP traits of the isolated strains are positively correlated with the PSBs abundance and the sample characteristics (soil and climate). The morphological and biochemical characteristics of the strain allowed us to identify around nine different bacterial genera, including *Bacillus*, *Pseudomonas*, *and Rhizobium*. The findings showed that bacterial communities, density, and potency are closely correlated to various edapho-climatic conditions such as temperature, precipitation, soil nutrient status, and soil texture. These findings could be used to improve an effective plant-PSBs system and increase agricultural output by taking into account their specific ecological traits and plant growth mechanisms.

## Introduction

Phosphorus (P) is the second most essential nutrient after nitrogen, required by plants for growth and development. The low availability of P in the soil is mainly due to the complexes it forms with other elements when applied, thus becoming unavailable to the plant without the help of microbes [1]. In acidic soil, P forms complexes with Al and Fe oxides, while in alkaline soil, it forms complexes with calcium [2]. Phosphorus solubility is mediated through the secretion of organic acids that lower pH, the chelation reaction of phosphorus-bound ions, and competition with phosphorus for adsorption sites in the soil. Beneficial microbes, such as P-solubilising bacteria (PSB), a group of the Plant Growth Promoting Rhizobacteria (PGPR) community, can promote growth through plant-essential metabolites production, including phytohormones and minerals. PSBs interest has grown significantly over the last three decades for two main reasons; first, there is a growing depletion of extractable P rocks [3], and second, an estimated 5.7 billion hectares of arable land around the world have insufficient free orthophosphate for optimal crop production [4]. For this reason, attention has recently been focused on the discovery of PSBs, e.g. the genera *Bacillus*, *Pseudomonas*, *Rhizobium*, *Enterobacter*, and *Burkholderia* which can reduce the use of chemical P fertilizers [5–7] by the production of organic acids which lowers the pH and helps convert non-assimilable divalent and trivalent forms of P into assimilable monovalent forms [8] while protecting plants from abiotic stresses under a wide range of climatic conditions. These rhizobacteria promote plant growth through direct and indirect plant growth promotion (PGP) mechanisms. Among these, auxin production, siderophores production, zinc solubilization, and P solubilization are desirable traits that are commonly suggested as mechanisms to promote plant growth by effective PGPs.

Soils contain diverse communities of microorganisms that interact with plants and respond to their specific physical and chemical characteristics. These microbial communities contribute to essential soil ecosystem services, including organic matter decomposition, nutrient cycling, disease control, and plant nutrition [9]. Climate models show that the Mediterranean region is a hot spot for climate change. The combined effects of decreasing precipitation and increasing temperatures make the Mediterranean region one of the most vulnerable ecosystems. Soil microbial communities are not randomly distributed but show spatial aggregation [10]. In particular, microenvironments can develop following long-term stress through adaptive evolution [11], which leads to soil microorganisms selection with distinct genetic and metabolic characteristics [12]. This adaptive evolution generates biodiversity in soil [13].

The occurrence, abundance, diversity, and bioactivity of PSB vary in different soils. The variation is attributed to the different soil properties including the nutritional conditions and physiochemical properties. However, PSBs are influenced by biotic or abiotic factors such as interactions with other microorganisms, agronomic activities, ecological conditions, and soil types [14]. Research has also shown that PSBs are largely dependent on some produced metabolites and their release rate [14]. Deiss et al. (2018) [15] determined that climatic variables (average annual precipitation and temperature) diversely regulate the soil inorganic and organic P pools in combination with edaphic variables mainly determined by parent material and soil formation factors. In addition, well-aerated soil is more conducive to rapid phosphorus solubilization than saturated wet soil. Lime and compost, used as soil amendments, also have positive effects on phosphate solubilization [14]. Amadou et al. (2020) [16].observed that soil amendment with biochar and manure, as well as the addition of inorganic mineral fertilizers, changed the soil properties, especially the NH^4+^ content, and this had an impact on the structure of the bacterial community above and below the soil (rhizosphere and root). Among the different abiotic stresses, low temperature is a major limitation to the growth, reproductive stage, and plant grain yield [7]. In addition, interactions between plants and microorganisms are influenced by temperature changes. Li et al. (2021) [17] show that on a continental and global scale, the population density of PSB is correlated with total P rather than pH and is influenced by environmental factors. It is, therefore, remarkable that positive relationships exist between soil PSB population density and available P, nitrate-nitrogen, and dissolved organic carbon in the soil, reflecting functional couplings between PSBs and microbes that drive the biogeochemical cycles of nitrogen and carbon [17].

Since PSBs can play a key role in enhancing the host plant’s ability to withstand stressful conditions. We hypothesized that the environmental conditions observed at the sampling sites might be correlated with the soil types, the expression levels of the different P-solubilising traits of the isolated bacteria, and, conversely, their potential to promote plant growth. Therefore, we addressed the question: are the performance and plant growth promotion mechanisms of rhizobacteria associated with the environmental conditions of their soil source? Considering the above facts, the present study focused on the several effects of climate and soil conditions on the abundance, potential, and diversity of PSBs in the rhizosphere of legumes in four dissimilar bioclimatic stations of Fez—Meknes regions, Morocco. In addition, to assess the potential impact of these factors on the capacity of PSBs, we tested the P solubilization efficiency of isolates, evaluate their plant’s growth trait potential, and assessed the impact of environmental conditions at each study site on the PSB strains. A better understanding of microbial consortium patterns is essential and becomes a prerequisite for the sustainable use of these biofertilizers in agriculture. The study results could provide an effective approach for the agronomic improvement of microbial inoculants to enhance soil P mobilization for plant growth either in intensive production or in agroecological applications in Morocco.

## Materials and methods

### Soil samples and isolation of phosphate solubilizing bacteria

**Fig 1:**
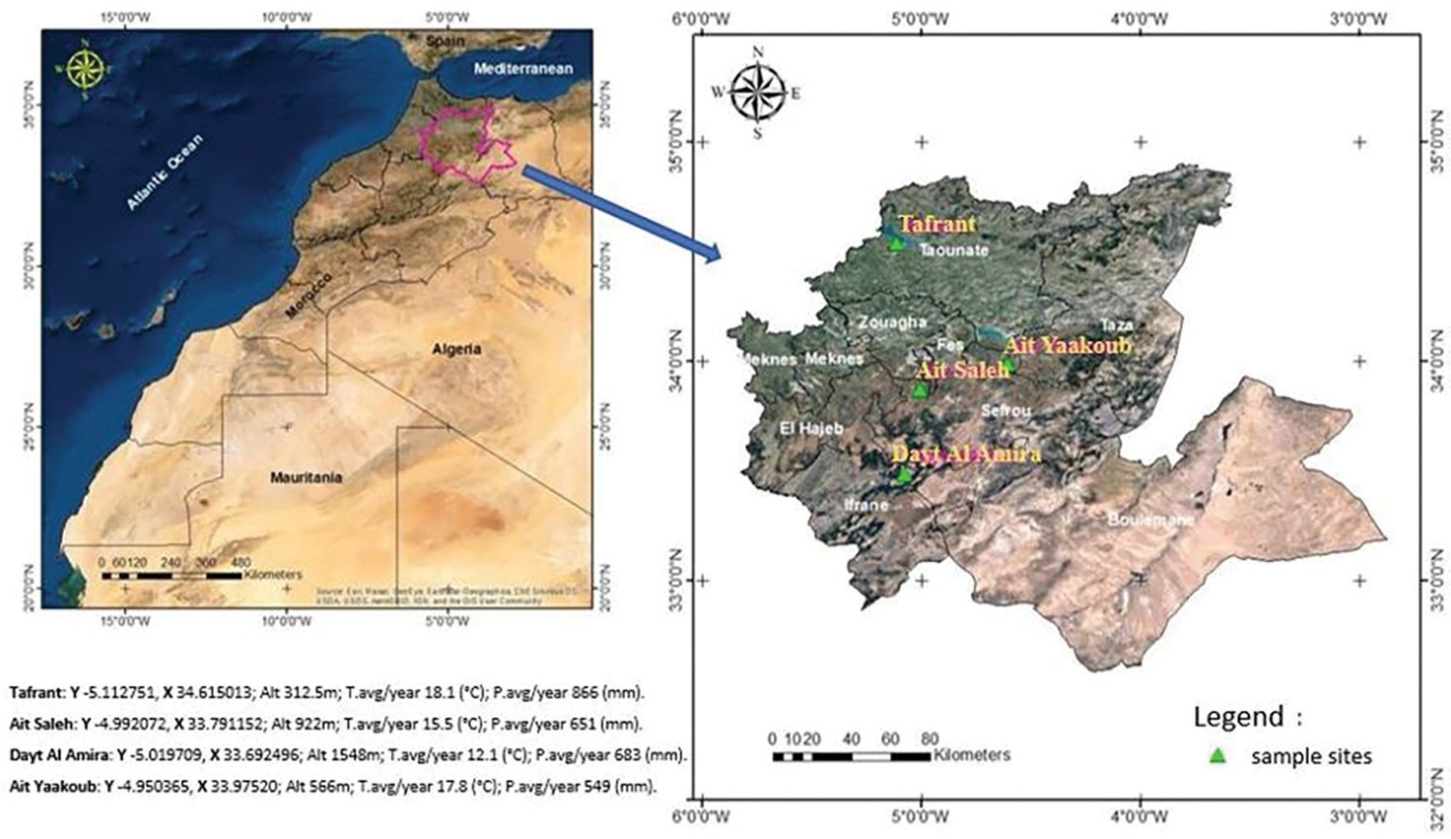
Soil sampling sites located at Fez—Meknes region (Morocco).

Soil samples were collected from cultivated and spontaneous plants from different rhizosphere sites of four dissimilar bioclimatic stations distributed in the sub-mountainous and central areas of the Fez—Meknes regions from December to January 2020 (Fig 1). As this is a simple soil sample that does not harm the environment, the regional authorities do not require us to obtain a permit or any legal obligation. Five samples are taken at each site, and the values at each site represent the average of the five samples. The selected fields from which samples are taken were under cultivation, and rhizosphere sampling was carried out after crop harvest at the end of wheat, barley, beans, peas, and beans planting season. From each sample, a part was consecrated to PSBs isolation.

Phosphate solubilizing bacteria were isolated on National Botanical Research in P medium (NBRIP) agar plates [18, 19]. To determine the efficiency of P solubilization, the PSB isolates were grown in an NBRIP agar medium supplemented with 5 g.L^-1^ TCP. After nine days of incubation at 28 ± 2°C, the colonies’ diameter and the visible halos were measured to calculate the P solubilization index (PSI) by the following formula (Eq 1):

$$PSI=\frac{CD+HD}{CD}$$ (1)

*CD: Colony Diameter*. *HD: Halo zone Diameter*.

### Sites description

Samples collected from the crop field correspond to four sites named Ayt Saleh, Ayt Yaakoub, Tafrant, and Dayt Amira. Sites samples description and bacteria analyses are described in Table 1. Soil physiochemical characterization such as organic matter, pH, conductivity, soil texture, available nitrogen, phosphorus, and potassium in the soil, soil micronutrient status, total mineral content, etc. was analysed using standard methods at the pedology department at the National School of Agriculture, Meknes, Morocco. Soils in these regions are classified as alkaline with medium P content. Soil samples from all stations have major variations in clay, silt as well as in sand content. The value of silt content is higher than that of clay and sand, except for the Tafrant site where the clay represents the major part. For heavy metal concentration analysis, representative soil samples were air-dried and sieved (<2 mm), and concentrations of Zn, Pb, Co, Cd, Ni and Cr were determined, after extraction with mineralization, by Inductively Coupled Plasma (ICP).

**Table 1:** Sites samples description.

Stations/Parameters	Tafrant	Ait Yaakoub	Dayt Al Amira	Ait Saleh
Coordinates	**Y** -5.112751, **X** 34.615013	**Y** -4.950365, **X** 33.975209	**Y** -5.019709, **X** 33.692496	**Y** -4.992072, **X** 33.791152
Altitude (m)	312.5	566	1548	922
T. avg/year (°C)	18.1	17.8	8.3	15.5
P. avg/year (mm)	866	549	683	651
pH	8.26	8.32	8.07	8.27
Sand (%)	15.3	21.6	8	31.9
Silt (%)	33.5	46.8	55.0	35.6
Clay (%)	51.2	31.5	37.1	32.6
Texture Triangle USDA	Clay	Clay Loam	Silty Clay Loam	Clay Loam

### Climatic study by emberger bioclimatic quotient

The current study has focused on the Emberger rainfall quotient to determine the climate type that governed each studied area. It is the ratio between precipitation amount and the average of thermal extremes (warmest—coldest months), all corrected by the extreme thermal amplitude (M—m). Emberger noted that thermal amplitude is a significant factor in plant species distribution, which leads to microbial species diversity [20, 21]. This approach places meteorological stations at the appropriate bioclimatic stage providing a better understanding of the Mediterranean region’s aridity and its effect on PSBs abundance in studied sites. The following formulas were used to calculate the Emberger quotient (Table 2).

**Table 2:** Emberger bioclimatic quotient formulas.

formulas	References	Coefficients
Q_2_ = 200*P/(M^2^-m^2^)	[20]	P: Annual rainfall in mm/m^2^/year. M: Maximum temperature of the hottest month in ^∘^K. m: Minimum temperature of the coldest month in ^∘^K.
Q_2_ = 3.43*P/(M-m)	[22]	
Q_2_ = 2000*P/(M+m+546.4)*(M-m)	[23]	M: Maximum temperature of the hottest month in ^∘^C. m: Minimum temperature of the coldest month in ^∘^C.

To investigate the effects of climatic and edaphic parameters on PSBs population density in the environmental samples, we analyzed the relationships between these parameters and PSBs population density using univariate linear regressions. In addition, the rainfall and temperature of each site were studied using an umbrothermal diagram to give more details on how PSBs abundance and diversity can be affected.

### Screening of bacterial plant growth promoting traits

#### Quantitative estimation of phosphate solubilization

To measure the solubilization capacity of isolates a liquid NBRIP medium supplemented with 5 g.L^-1^ RP alone and 5 g.L^-1^ TCP alone was used. The amount of assimilable P content was carried out by the ascorbic acid colourimetric method [24] after seven days of incubation and with few modifications. A standard range of KH_2_PO_4_ (concentration from 0 to 1 mg.L^-1^) was used to calculate P content. To identify PSB genera, we extended the morphological and biochemical tests conducted by Janati et al. (2022) [19]. In this paper, we examine the preliminary screening of PSB isolates and investigate the species affiliation of each potential isolate for phenotypic characterization of phosphate solubilizing bacteria based on growth in a different medium, according to Bergey’s textbook (Systematic Bacteriology, 2nd ed., vol.5, eds., 2012).

#### Analysis of auxin production

Auxin production of PSBs was determined according to the method of [25] with some modifications. The test strains were cultured into L-tryptophan-containing LB liquid medium (addition of 1 L LB + 0.1 g L-tryptophan) and incubated on a shaker at 180 rpm at 28 ± 2°C for 4 days. The broth was centrifuged at 6800 × g for 10 min. The supernatant was isolated and 0.1 mL was mixed with 0.1 mL Salkowski’s reagent (4.5 g FeCl_3_ and 587.4 mL of 98% H_2_SO_4_, solution) and kept in the dark at room temperature for 30 min. The development of a pink-red colouration indicates the production of auxins. The absorbance of each sample was measured with a spectrophotometer at 530 nm, and auxin concentrations were determined based on a standard range prepared with standard solutions ranging from 0 to 50 μg.mL^-1^ auxins.

#### Siderophores production activity assays

The culture on agar medium containing chromium azurol S (CAS) allows the detection of the secretion of siderophores by the microorganisms. The principle is that the culture medium initially has a blue colour due to the Iron/CAS/HDTMA complex which turns orange-red following the displacement of iron by the siderophores produced by the microorganism. All the test strains were inoculated into TSA liquid medium and were shaken at a 180 rpm incubator for 2 days at 28 ± 2°C. The fermentation broth was centrifuged at 6800 × g for 10 min. A solution consisting of 60.5 mg CAS in 50 mL of deionized water was amended with 10 mL FeCl_3_.6H2O solution. Then, 72.9 mg HDTMA (hexadecyltrimethylammonium bromide) dissolved in 40 Ml of deionized water was added to CAS to a final volume of 100 mL [26, 27]. Four millilitres from the prepared CAS solution were mixed thoroughly with 4 mL of the culture supernatant, followed by incubation for 1 h. The absorbance of each sample was measured with a spectrophotometer at 630 nm. Siderophores production by bacterial strains was evaluated as the percentage of siderophores units (Eq 2).


SUs=[(Ar‐As)/Ar]×100 (2)

*Ar* *=* *absorbance of reference (CAS reagent) and As = absorbance of the sample*.

#### Qualitative test for zinc solubilising bacteria

The zinc-solubilizing ability of these strains was evaluated in mineral salt medium (MSM: NaCl 1g, CaCl_2_ 0.1 g, MgSO_4_ 0.5 g, KH_2_PO_4_ 1 g, K_2_HPO_4_ 1 g, yeast extract 4 g, agar 15 g in 1 L, and pH was maintained at 7.2). Different sources of insoluble zinc salts like zinc oxide (1.244 g/L = 15.23 mM), zinc phosphate (1.9882 g.L^-1^ = 5.0 mM), zinc carbonate (1.728 g.L^-1^ 5.2 mM), and zinc sulfide (1.124 g.L^-1^ 11.54 mM) at a final concentration of 0.1% were added in the medium individually and autoclaved at 121°C for 30 minutes [28]. A loop was full of bacterial overnight growth in nutrient broth was spread on an MS medium. Plates were incubated at 30°C for 7 days. Strains showing a clear zone around the colony are considered zinc-solubilizing strains [29]. The Zinc solubilizing index (ZSI) was calculated as the following formula (Eq 3).


ZSI=(halo+colony)/colonydiameters (3)

#### ACC deaminase activity

The isolates were checked for their ability to utilize ACC as an N source by assessing their growth in DF minimal salts medium [30] supplemented with 3 mM ACC. The ACC deaminase activity of the isolates was measured using a spectrophotometer that measured the absorbance at 540 nm, the α-ketobutyrate produced from the enzymatic cleavage of ACC through ACCD as discussed previously by Honma & Shimomura, (1978) [31]. The α-ketobutyrate produced by this reaction is estimated by comparing the absorbance at 540 nm of a sample to a standard curve of α-ketobutyrate ranging between 0.1 and 1.0 mM [32].

### Identification of phosphate-solubilizing bacteria

Following Bergey’s Manual (Systematic Bacteriology, 2nd ed., vol. 5, eds., 2012), the preliminary screening for PSB isolates was performed to investigate species affiliation of each potential isolate based on growth in a different medium; King A and King B medium to identify pseudomonas [33], Yeast extract mannitol agar medium (YEMA) to select rhizobium [34], Mannitol Salt Agar medium (MSA) to identify staphylococcus [35], Eosin methylene blue medium (EMB) to identify E. Coli [36], De Man, Rogosa and Sharpe medium (MRS) to identify lactic bacteria [37]. Motility test, Gram staining, catalase, gelatinase, and urease activities, starch glucose, lactose as a source of carbon, citrate utilization and H_2_S production were performed.

### Statistical analyses

The region map was prepared using the SIG software. Data were analyzed using SPSS software, and results were expressed as the means ± standard deviation of three replicates. Data were examined by ANOVA I analysis, and mean comparison was performed by Duncan’s multiple range test at p ≤ 0.05. Principal Component Analysis (PCA) was performed to give a summarising view of obtained results, how soil and environmental characteristics influenced isolates biological attributes, and to determine which inter-related parameters most influenced isolates P solubilizing potential of the genera diversity indices were calculated using PAST free software version 3.

## Results

### Soil samples analysis

One hundred sixty-four PSBs strains were obtained from the rhizosphere of legumes, cereals, and spontaneous plants based on morphologically different colonies and remarkable halo solubilization formed in the NBRIP-TCP medium. Fifty-one strains showed different capacities for P-solubilization and soluble P concentration in NBRIP-TCP and NBRIP-RP mediums and were named as PSBs. Ait Saleh region showed the highest PSBs load of 3.16x10^4^ (Cells.Kg^-1^ of soil). The value of air-filled porosity present in the studied soils indicates adequate pore distribution and continuity; Dayt Al Amira and Ait Saleh present the highest value of 15%. The result confirms that Ait Saleh has the maximum organic matter (5.64%) and permeability (29 mm/hr). On the other hand, the Dayt Al Amira site showed the highest amount of P_2_O_5_, K_2_O, and MgO (98.7 mg.Kg^-1^, 990.7 mg.Kg^-1^, and 1213.3 mg.Kg^-1^ respectively), however, Tafrant site has the highest value of CaO with 9450 mg.Kg^-1^. There was a positive correlation between total N and mineral N in the studied regions, Ait Yaakoub and Dayt Al Amira have the highest mineral N value (8.9 and 8.2 mg.Kg^-1^), respectively, against 0.3% of total N, followed by Ait Saleh and Tafrant. In terms of soil pH, it was close to neutral or slightly basic (pH = 8.23 on average), and the soil was moderately calcareous. Cation exchange capacity ranged between 31.6 and 41 cmol.kg^-1^. Maximum electrical conductivity, reflecting soil salinity, was recorded in the Ait Yaakoub sample at 300.33 (ds.m^-1^).

The average percentage of nutrient parameters in the soil showed important mean values. Agricultural legume soils analyzed for physicochemical characteristics revealed that Dayt Al Amira has the highest mineral content region with remarkable values of Al, Ca, Mg, and P (Table 3). We noted that this region has a silty clay loam texture with just 8% of sand. Moreover, the Dayt Al Amira region has minimal values of total limestone (8.3%) and active limestone (2.5%). In addition, from the mineralogical compositions of different sites analyzed with ICP, we notice that Al, Ca, Fe, K, Mg, Na, and P are the most abundant minerals in various sites’ distribution.

**Table 3:** PSBs load and Physico-chemical analysis of the soil studied sites.

Sites/Parameters	Tafrant	Ait Yaakoub	Dayt Al Amira	Ait Saleh
PSBs load (Cells.Kg^-1^)	59.5	198	1040	31600
Bulk Density (%)	1446	1459	1464	1462
Air Filled Porosity (%)	13	14	15	15
Specific Gravity (kg.m^-3^)	2.81	2.74	2.65	2.67
Permeability (mm.hr^-1^)	26	28	28	29
P_2_O_5_ (mg.Kg^-1^)	10	46.7	98.7	23.3
K_2_O (mg.Kg^-1^)	432	846.3	990.7	373
CaO (mg.Kg^-1^)	9450	8550	5250	6375
MgO (mg.Kg^-1^)	410.7	854	1213.3	917.3
P (mg.Kg^-1^)	0.04	0.02	0.04	0.03
K (mg.Kg^-1^)	0.21	0.21	0.46	0.26
Fe (mg.Kg^-1^)	1.29	0.62	1.24	0.94
Al (mg.Kg^-1^)	1.02	0.79	1.19	0.95
Ca (mg.Kg^-1^)	2.18	0.54	2.14	1.79
Mg (mg.Kg^-1^)	0.39	0.17	1.41	0.89
Na (mg.Kg^-1^)	0.44	0.68	0.84	0.97

Ag, As, Be, Bi, Co, Nb, Ni, Pd, Pt, Se, Zr < 0.001

B, Ba, Cd, Cr, Cu, Li, Mn,Mo, Pb, Sb, Si, Sn, Sr, Ti, V, Zn < 0.01

### Climatic study using emberger bioclimatic quotient

**Fig 2:**
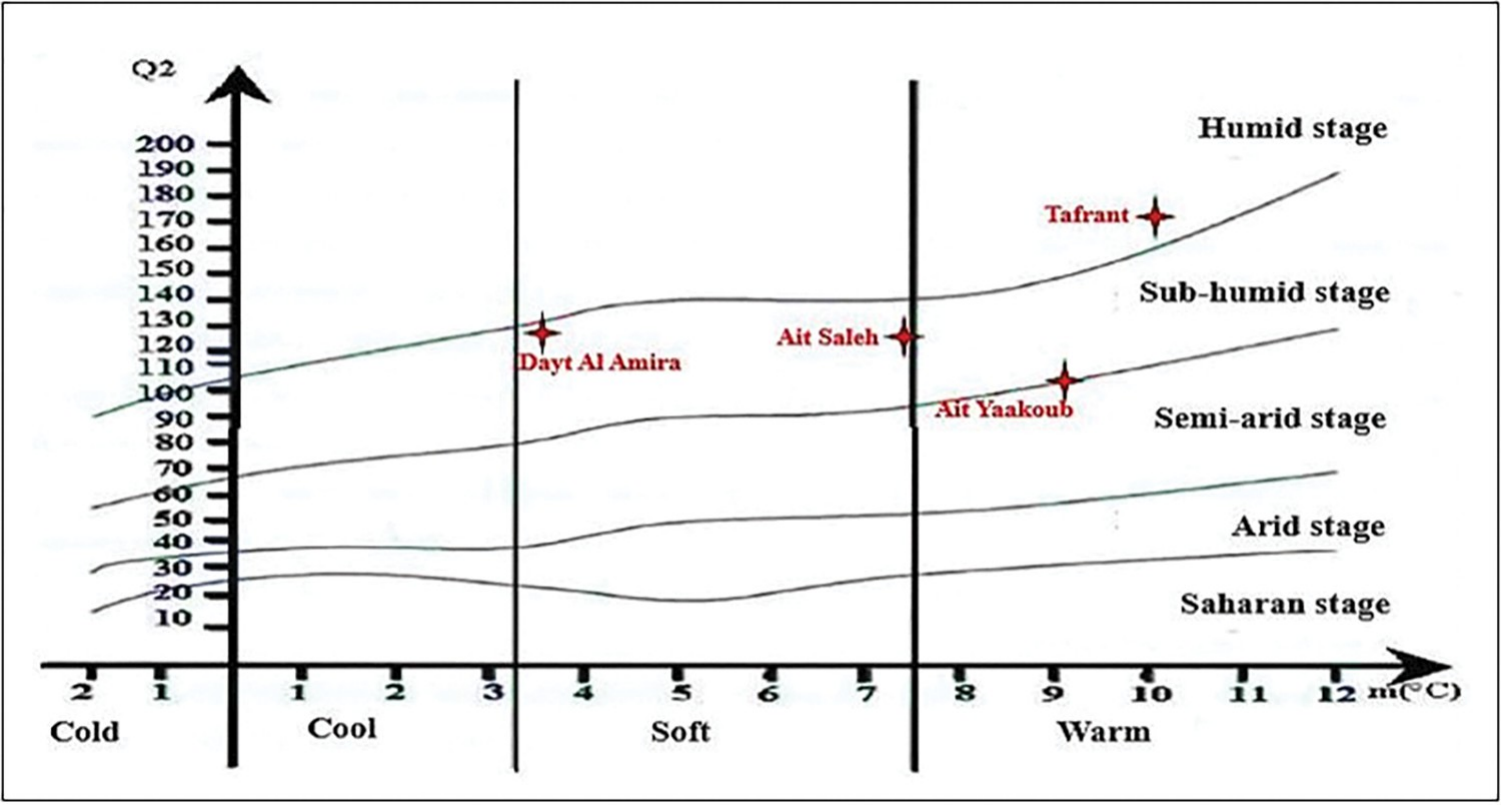
Emberger climatogram of soil studied sites.

The regions studied are characterized by different thermal and water profiles. Rainfall-thermal quotients (Q_2_) for the different regions using three methods show that all formulas give approximately the same results. The value of Q_2_ using Stewart’s formula differs only slightly from the Q_2_ developed by Emberger and Mokhtari (Table 4). Emberger subdivided the climatic zone into characteristic zones combining the rainfall-thermal quotients Q_2_ obtained and the minimum temperatures of the coldest month (m) (limiting factor). The Q_2_ values plotted on the Emberger diagram illustrate the shift in Fig 2.

**Table 4:** Rainfall-thermal quotients (Q_2_) of the four studied sites.

Regions/Q_2_	Tafrant	Ait Yaakoub	Dayt Al Amira	Ait Saleh
Q_2_ Emberger	172.52	101.24	127.71	126.35
Q_2_ Stewart	172.70	101.24	125.28	125.45
Q_2_ Mokhtari	172.49	101.22	127.68	126.33
Average	172.57	101.23	126.89	126.04

We noted that Dayt Al Amira and Ait Saleh sites have close values of Q_2_ (126 approximately). However, the Tafrant site has the most significant value and the Ait Yaakoub site has the minimum value. These results give an idea of the PSBs distribution in these regions given that the Ait Saleh and Dayt Al Amira sites have the most important PSBs load respectively.

The climatogram analysis reveals that the Tafrant site is the only site subjected to the humid bioclimatic stage in warm winter with Q_2_ being approximately 172.57. On the other hand, the Ait Yaakoub site is placed at the limit of sub-humid and semi-arid climate with warm winter and Q_2_ close to 101.23, whereas the two other sites Dayt Al Amira and Ait Saleh are in sub-humid climate in soft winter, with Q_2_ close to 126.89 and 126.04, respectively.

Furthermore, the umbrothermal diagram shows that the Tafrant site has the most precipitation but is countered by high temperatures, such as the Ait Yaakoub site, which exceeds 17.8 degrees Celsius on an annual basis. This is not the case for the Ait Saleh and Dayt Al Amira sites, which have significant rainfall and moderate to low temperatures respectively.

**Fig 3:**
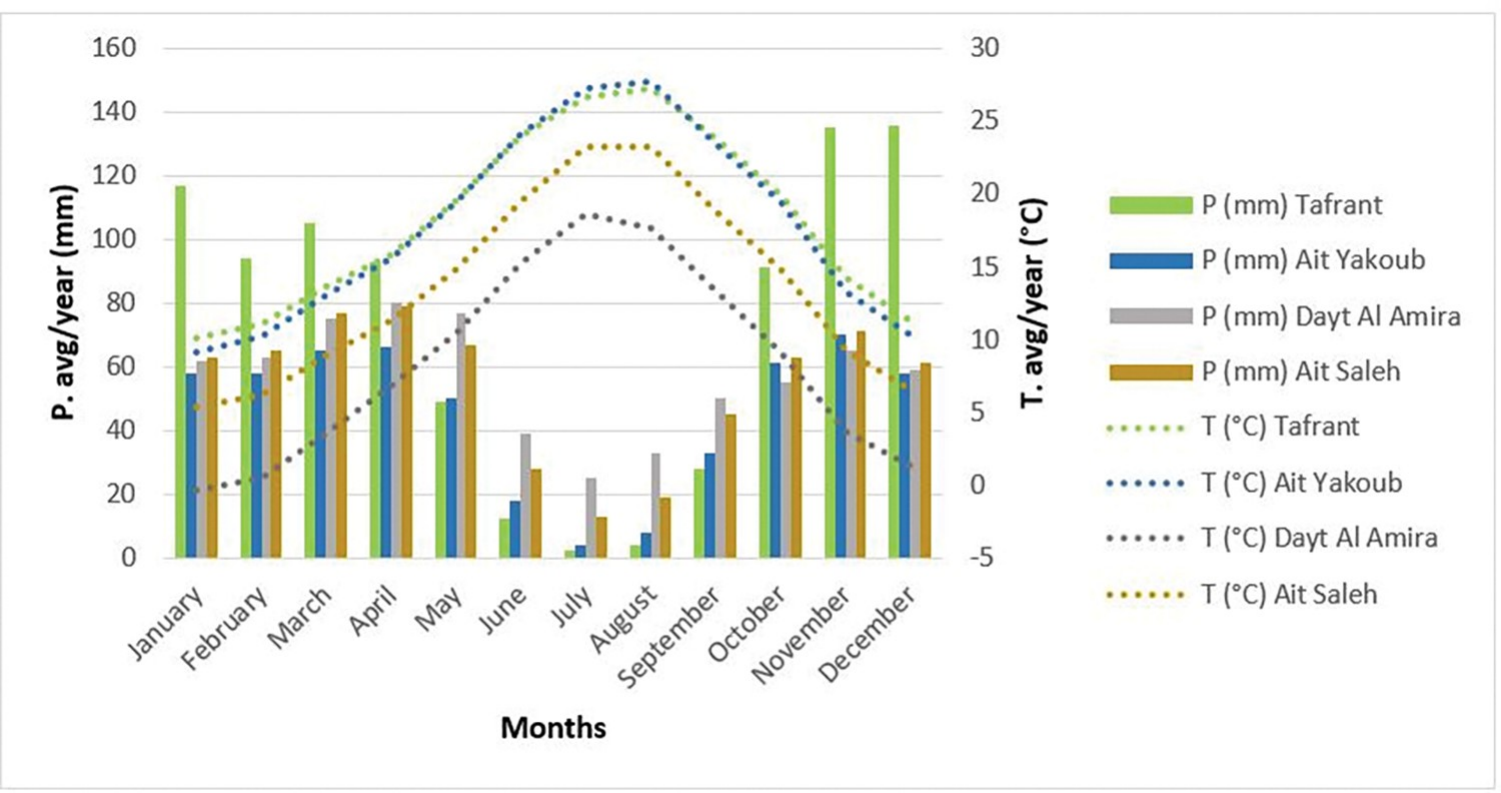
Umbrothermal diagram of the studied sites.

The lowest average rainfall in all regions makes July the driest month of the year, with only 2 mm in Tafrant, 4 mm in Ait Yaakoub, 13 mm in Ait Saleh, and 25 mm in Dayt Al Amira. While an average of 136 mm makes December the month with the highest rainfall in Tafrant. Although, Ait Yaakoub record’s rainfall is recorded in November with an average of 70 mm. However, in Ait Saleh and Dayt Al Amira, April has the highest rainfall of the year with an average of 79 mm and 80 mm, respectively (Fig 3). This figure is depicting a negative correlation among temperature and PSB load. We note that the high microbial load at the Ait Saleh site may also be influenced by other parameters studied. The strong negative correlation between rainfall and microbial load is clearly observed at the Tafrant site. On the other hand, moderate rainfall favours microbial load at the Ait Saleh and Dayt Al Amira sites.

### Screening of bacterial plant growth promoting traits

#### Phosphate solubilizing assessment

**Fig 4:**
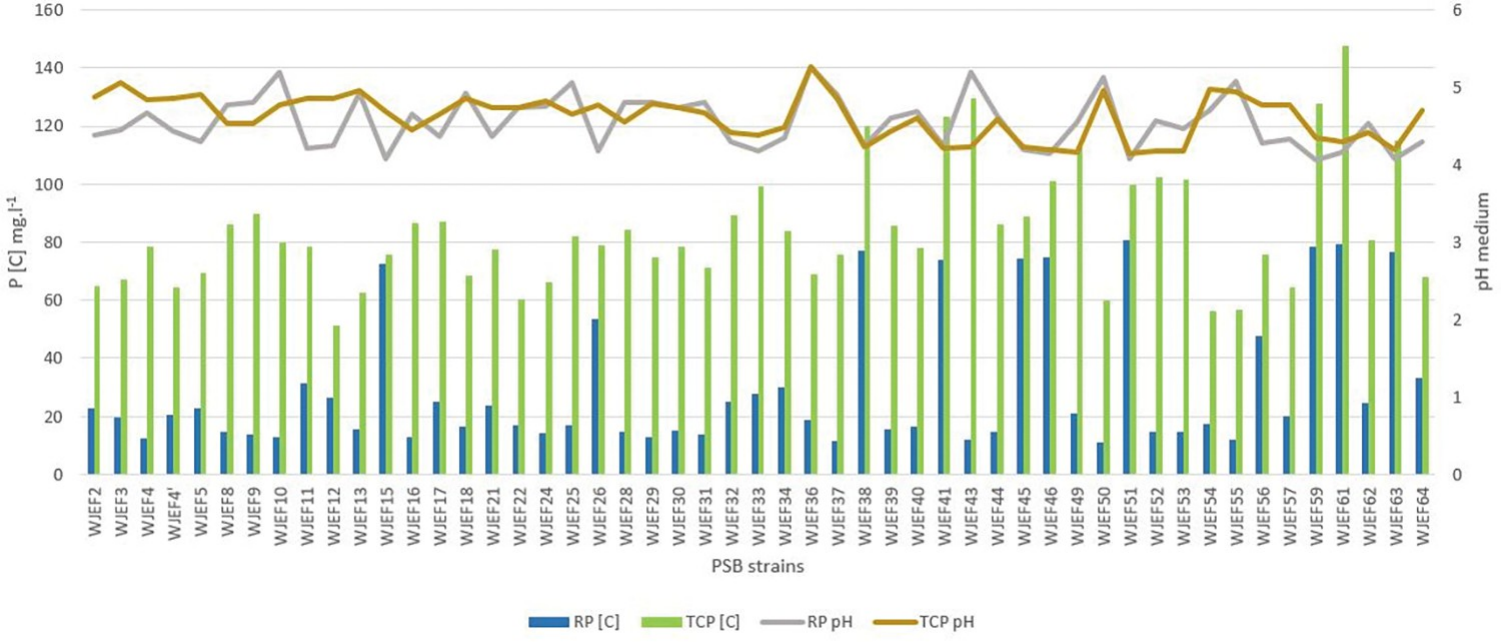
The average concentration of RP and TCP solubilization accompanied with pH variation in NBRIP liquid medium of all studied strains after nine days of inoculation.

Results revealed that all isolated strains from studied regions were able to produce halo-forming colonies on the NBRIP-TCP amended solid medium. Although, there was a significant difference in all tested strains. The solubilization halo zone ranged from 1.1 cm to 4.2 cm, and the appearance of the halo zone in all strains was noticed after the 4^th^ day of incubation. Isolate WJEF8 shows a maximum solubilization index with PSI = 3.84 [19] (Table 5). Furthermore, we noted that an increase in incubation time leads to an improvement in the halo zone size of each isolate, with a correlation between incubation time and halo zone size. The isolated strains were further screened to confirm their inorganic phosphate solubilizing efficiency on TCP- and RP-NBRIP broth medium. The results of 9 days of solubilization experiments and pH variation are presented in (Fig 4). The obtained results show that when TCP was used in NBRIP broth as the only P source, the concentration of soluble phosphorus released by the isolates in each site varied between 71.71 mg.L^-1^ and 94.54 mg.L^-1^ accompanied by significant variations between the different isolates (p ≤ 0.05). Maximum TCP solubilization was observed for the WJEF61 strain. On the other hand, the concentration of soluble P in the RP-NBRIP medium in each site varied between 18.69 mg.L^-1^ and 40.43 mg.L^-1^ accompanied by significant variations between the different isolates (p ≤ 0.05). The maximum solubilization of RP was observed for the WJEF51 strain. These isolates were found to solubilize TCP more than RP and the most occurred ones were in the Ait Saleh site. The results of the experiments indicated that P solubilization varied according to the microbial isolates and the type of insoluble phosphate (TCP or RP). The solubilization of TCP and RP in the NBRIP liquid medium by the tested strains was accompanied by a significant decrease in pH.

**Table 5:** The average PSI in NBRIP-TCP solid medium of all studied strains of each sampling site.

Sites	Strains	PSI
Tafrant	WJEF2	2.29
	WJEF3	2.47
	WJEF4	2.31
	WJEF4’	2.56
	WJEF5	2.50
	WJEF8	3.84
Ait Yaakoub	WJEF9	1.90
	WJEF10	2.15
	WJEF11	2.54
	WJEF12	2.38
	WJEF13	2.07
	WJEF15	2.46
	WJEF16	2.22
	WJEF17	2.29
	WJEF18	2.36
Dayt Al Amira	WJEF21	2.09
	WJEF22	2.75
	WJEF24	2.85
	WJEF25	2.90
	WJEF26	2.67
	WJEF28	2.67
	WJEF29	1.40
	WJEF30	2.57
	WJEF31	2.15
	WJEF32	2.33
	WJEF33	2.50
	WJEF34	2.33
	WJEF36	2.31
	WJEF37	2.39
Ait Saleh	WJEF38	2.30
	WJEF39	2.50
	WJEF40	2.78
	WJEF41	2.27
	WJEF43	2.20
	WJEF44	2.09
	WJEF45	2.30
	WJEF46	2.27
	WJEF49	2.50
	WJEF50	2.33
	WJEF51	2.33
	WJEF52	2.40
	WJEF53	2.33
	WJEF54	2.22
	WJEF55	2.11
	WJEF56	2.33
	WJEF57	2.30
	WJEF59	2.30
	WJEF61	2.14
	WJEF62	2.64
	WJEF63	2.33
	WJEF64	2.17

The pH of the medium inoculated with the selected strains decreased significantly from 4.88 to 4.47 (TCP) and 4.67 to 4.50 (RP), respectively, after nine days of incubation. Based on the one-way analysis of variance (ANOVA), we noted a significant difference between the solubilization of RP and TCP within the four sites. It is interesting to mention that the Ait Saleh site has the highest solubilization potential, taking into account that this location has the maximum PSBs load.

#### Analysis of auxins production

**Fig 5:**
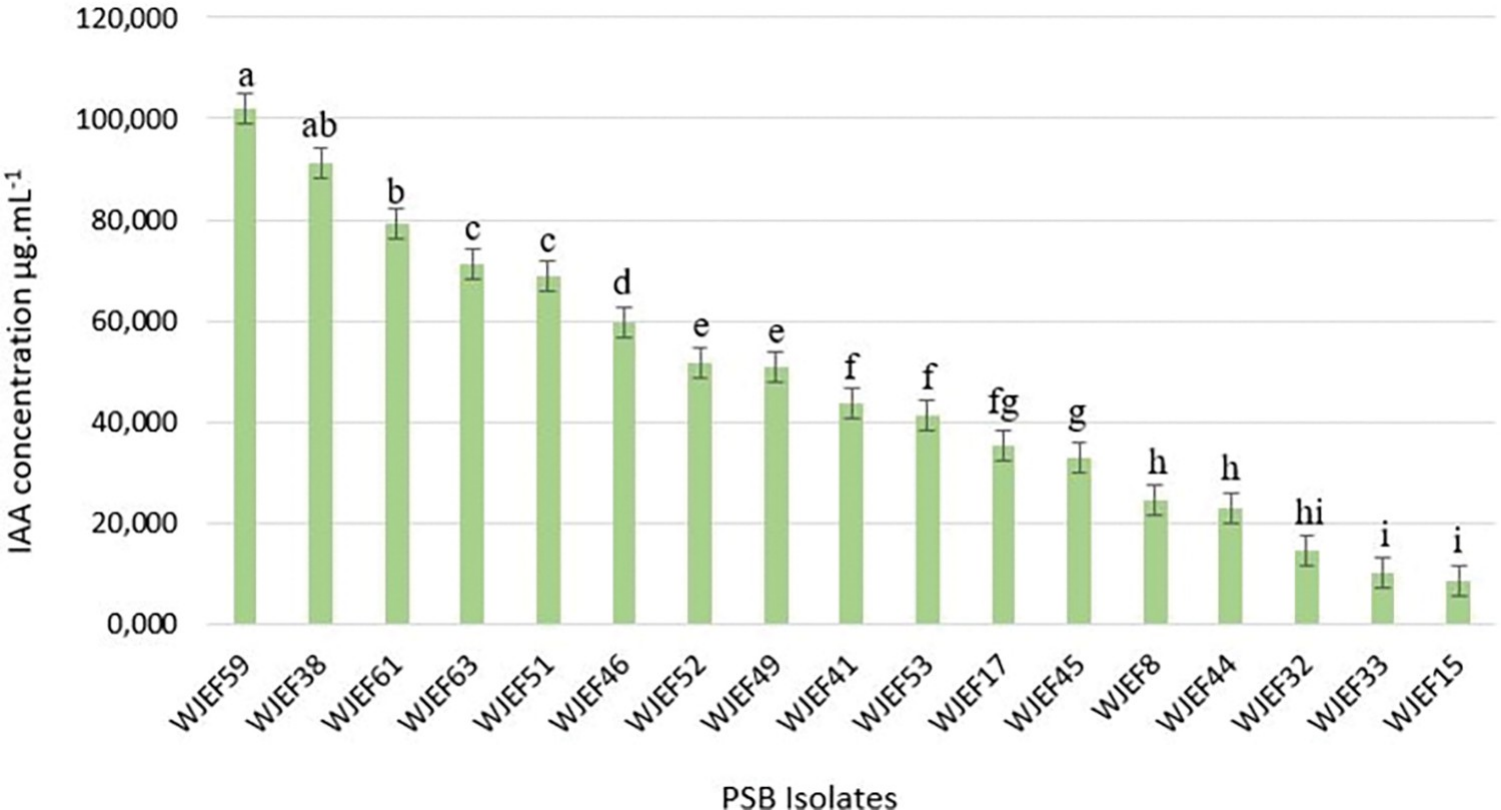
Auxins concentration levels produced by bacterial isolates. The different letters indicate a significant difference (p<0.05), Mean values (n = 3).

The data obtained on auxins production showed that from 51 isolates approximately 33.34% can produce auxins (Fig 5). It was observed that the isolates produce maximum auxins in the medium, by giving intense pink colour with the highest absorbance expressed as an optical density (OD) value. These isolates showed varying rates of production from 101.87 μg.mL^-1^ to 8.426 μg.mL^-1^. We emphasize that the Ait Saleh site is the source of 70.59% of the bacteria that produce auxins, in which WJEF59 has the highest auxins concentration of 101.87 μg.mL^-1^.

#### Analysis of siderophores production activity

**Fig 6:**
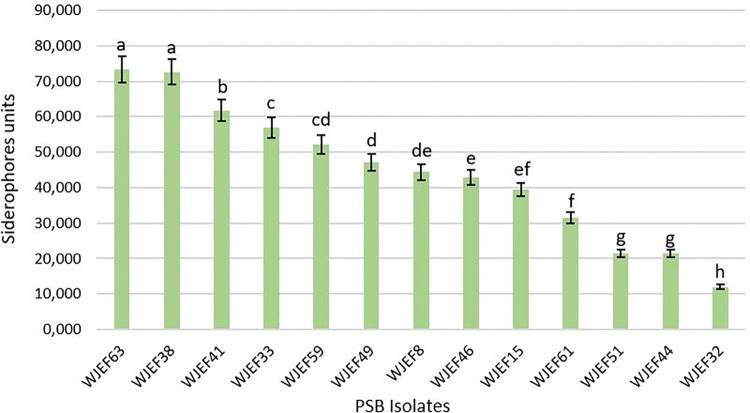
Siderophores levels produced by bacterial isolates. The different letters indicate a significant difference (p<0.05), Mean values (n = 3).

Based on the data collected on siderophores production, out of 51 isolates, approximately 25.49% can produce siderophores (Fig 6). These isolates produced between 12.06 and 72.26 Units. Like auxins production, the Ait Saleh site also contains the bacterial strains that produce siderophores accounting for 69.23% of the total bacteria isolated, where WJEF63 has the highest value of 73,26 Units.

#### ACC deaminase activity

From the 51 PSB isolates, five (WJEF15, WJEF26, WJEF38, WJEF46, and WJEF63) were assessed to be ACC deaminase producers in which values ranged from 0.27 to 0.58 nmol α-ketobutyrate mg/protein/h) (Table 6). The highest ACC deaminase activity was exhibited by bacterial strain WJEF38 from the Ait Saleh site (0.58 nmol α-ketobutyrate mg/protein/h).

**Table 6:** ACC deaminase activity by PSB strains. Data represent the mean ± of three independent replicates. Values with the same superscript letter were not significantly different according to the Duncan test performed (P < 0.05).

Strains	WJEF15	WJEF26	WJEF38	WJEF46	WJEF63
ACC deaminase activity	0.56^a^	0.27^c^	0.58^a^	0.49^b^	0.55^a^

#### Qualitative test for zinc solubilising bacteria

The results on Zn-solubilization revealed that approximately 23.53% of 51 isolates can solubilize four sources of Zn: zinc oxide, zinc phosphate, zinc carbonate, and zinc sulfide. When compared to other compounds, the isolates were found to be more efficient to solubilize zinc from zinc phosphate. Bacteria from the Ait Saleh region dominate this study, accounting for 75% of all zinc-solubilizing bacteria. The halo zone of solubilization ranged from 14 mm to 31 mm, whereas ZSI ranged from 2.22 to 4, (Table 7). We noted that all zinc solubilizing bacteria have different preferences for zinc compounds, except WJEF59, which has a high potential to solubilize any zinc source, although this isolate is from the Ait Saleh site.

**Table 7:** Zinc-solubilization index of PSB isolates after 7 days on Tris-minimal medium supplemented with insoluble zinc compounds; values followed by the same letter a, b, c, d, e, f, g, h, i, in the same column show no statistical significant differences (p < 0.05). Mean values (n = 3).

Isolates	Zinc oxide	Zinc phosphate	Zinc carbonate	Zinc sulphide
WJEF8	2,44 ± 1.578^b^	2,28 ± .989^a^	3,10 ± .325^g^	3,00 ± .268^e^
WJEF15	3,40 ± .856^f^	2,64 ± 1.061^c^	2,42 ± 1.035^c^	2,41 ± 0.897^a^
WJEF17	2,73 ± 1.247^c^	2,64 ± 1.237^c^	2,34 ± 1.449^b^	2,44 ± 1.268^a^
WJEF33	2,35 ± .707^a^	2,92 ± .33^e^	2,46 ± .975^c^	3,08 ± .745^e^
WJEF38	3,27 ± .865^e^	2,53 ± 1.323^b^	2,79 ± .69^e^	3,56 ± .33^f^
WJEF41	3,44 ± .424^f^	3,20 ± .268^f^	2,53 ± .865^d^	2,64 ± 1.65^b^
WJEF44	3,09 ± .749^d^	3,30 ± .231^f^	2,92 ± 1.138^f^	2,85 ± .89^d^
WJEF46	2,38 ± 1.106^a^	2,77 ± .84^d^	2,81 ± .865^e^	3,09 ± .747^e^
WJEF49	3,30 ± .56^e^	3,82 ± .19^h^	2,53 ± 1.332^d^	2,46 ± .236^a^
WJEF59	3,88 ± .495^h^	4,00 ± .424^i^	2,92 ± 1.055^f^	3,56 ± .06^f^
WJEF53	2,64 ± .935^c^	3,00 ± .14^e^	2,22 ± 1.543^a^	2,77 ± .115^c^
WJEF51	2,50 ± 1.138^b^	3,56 ± .882^g^	2,91 ± .995^f^	2,40 ± 1.597^a^

#### Identification of phosphate-solubilizing bacteria

From one hundred and sixty-four bacterial isolates, fifty-one strains isolated from four separate sites representing different colony types were prepared for phenotypic identification. The morphological characteristic of the selected isolates showed that most bacterial colonies were entire, yellow, viscid, rod, and coccobacilli shapes. Except for fifteen isolates gram reaction indicates that all strains are gram-negative. In addition, all selected isolates were positive for cell motility test and catalase activity. Only one isolate from Ait Yaakoub and one isolate from Ait Saleh can use citrate for growth. Whereas the majority of isolates that can produce H_2_S belong to the Ait Saleh site In this study, nine bacterial genera were identified based on different tests performed in specific media for the phenotypic characteristics, namely *Pseudomonas*, *Rhizobium*, *Clostridium*, *Serratia*, *Proteus*, *Burkholderia*, *Staphylococcus*, *Streptococcus*, and *Bacillus* (Tables 8 and 9).

**Table 8:** Morphological and biochemical identification of the isolated strains.

Sites	PSB Isolates / Characteristics	Genera	Margin	Colour	Texture	Shape	Arrangement	Gram staining	Catalase	Motility	GPA (Glucose)	GPA (Lactose)	Citrate utilisation	Starch utilisation	Gelatine hydrolysis	Urea hydrolysis	King A medium	YEM medium	MSA medium	H_2_S Production
Tafrant	WJEF2	Ps	E	Y	V	R	CR	-	+	+	+	+	-	+	+	-	+	-	-	-
	WJEF3	Ps	E	Y	V	R	S	-	+	+	+	+	-	+	+	-	+	-	-	-
	WJEF4	Str	E	Y	V	R	CR	+	+	+	+	+	-	+	+	+	-	+	-	-
	WJEF4’	Bu	E	Y	M	CB	S	-	+	+	+	+	-	-	+	-	+	+	-	-
	WJEF5	Cl	E	Y	V	R	SP	+	+	+	+	+	-	-	-	-	+	+	-	-
	WJEF8	Rh	E	W	V	C	S	-	+	+	+	+	-	+	+	-	-	+	-	-
Ait Yaakoub	WJEF9	Cl	E	Y	D	R	CR	-	+	+	+	+	-	-	-	-	-	+	-	-
	WJEF10	Rh	E	W	V	CB	S	-	+	+	+	+	+	+	+	-	-	+	-	-
	WJEF11	B	E	Y	V	R	CR	+	+	+	+	+	-	+	+	-	-	+	-	-
	WJEF12	St	E	Y	M	R	CR	+	+	+	+	+	-	+	-	-	-	-	+	-
	WJEF13	Ps	E	Y	M	CB	S	-	+	+	+	+	-	+	-	-	+	+	-	-
	WJEF15	B	E	Y	V	R	S	+	+	+	+	+	-	+	+	-	-	+	-	-
	WJEF16	Se	E	Y	D	CB	S	-	+	+	+	+	-	-	+	-	-	+	-	+
	WJEF17	Ps	E	Y	V	CB	S	-	+	+	+	+	-	+	-	-	+	+	-	-
	WJEF18	B	E	Y	V	R	CR	+	+	+	+	+	-	+	+	-	-	+	-	-
Dayt Al Amira	WJEF21	St	E	Y	M	R	SP	+	+	+	+	+	-	+	-	-	+	-	+	-
	WJEF22	St	E	Y	V	R	CR	-	+	+	+	+	-	+	-	-	+	-	+	+
	WJEF24	Ps	E	Y	V	R	CR	-	+	+	+	+	-	+	+	-	+	+	-	-
	WJEF25	Rh	I	Y	M	C	S	-	+	+	+	+	-	+	+	-	-	+	-	-
	WJEF26	Ps	E	Y	V	R	CR	-	+	+	+	+	-	+	+	-	+	+	-	-
	WJEF28	Ps	E	Y	V	R	CR	-	+	+	+	+	-	+	+	-	+	+	-	-
	WJEF29	B	E	Y	M	R	S	+	+	+	+	+	-	+	+	-	+	+	-	-
	WJEF30	B	I	Y	D	R	CR	+	+	+	+	+	-	+	+	-	-	+	-	-
	WJEF31	Ps	E	Y	V	R	CR	-	+	+	+	+	-	+	-	-	-	+	-	-
	WJEF32	Rh	E	Y	V	R	CR	-	+	+	+	+	-	+	+	-	-	+	-	-
	WJEF33	Rh	E	Y	V	R	CR	-	+	+	+	+	-	+	+	-	-	+	-	-
	WJEF34	B	E	Y	V	R	CR	+	+	+	+	+	-	+	+	-	-	+	-	-
	WJEF36	Rh	E	Y	M	R	CR	-	+	+	+	+	-	+	+	-	-	+	-	+
	WJEF37	Pr	E	OW	V	R	S	-	+	+	+	+	-	+	+	+	+	+	-	-
Ait Saleh	WJEF38	St	I	Y	M	C	SP	+	+	+	+	+	-	-	-	-	-	+	+	+
	WJEF38	St	I	Y	M	C	SP	+	+	+	+	+	-	-	-	-	-	+	+	+
	WJEF39	Ps	E	Y	V	R	S	-	+	+	+	+	-	+	+	-	+	-	-	-
	WJEF40	Ps	E	Y	D	R	CR	-	+	+	+	+	-	+	+	-	+	-	-	-
	WJEF41	St	I	OW	M	CB	S	+	+	+	+	+	-	+	-	-	-	+	+	-
	WJEF43	Se	E	OW	V	R	CR	-	+	+	+	+	-	+	+	-	-	+	-	+
	WJEF44	B	I	Y	M	R	CR	+	+	+	+	+	-	+	+	-	-	+	-	-
	WJEF45	B	E	Y	V	C	S	+	+	+	+	+	-	+	+	-	-	+	-	-
	WJEF46	Rh	I	OW	M	CB	S	-	+	+	+	+	+	+	-	-	-	+	-	+
	WJEF49	Ps	E	Y	M	R	CR	-	+	+	+	+	-	+	+	-	+	-	-	-
	WJEF50	Pr	E	Y	V	R	CR	-	+	+	+	+	-	+	+	+	+	+	-	-
	WJEF51	Rh	E	OW	V	CB	SP	-	+	+	+	+	-	+	-	-	-	+	-	+
	WJEF52	Pr	E	OW	V	CB	S	-	+	+	+	+	-	-	-	+	-	+	-	+
	WJEF53	Rh	I	OW	V	CB	S	-	+	+	+	+	-	+	-	-	-	+	-	-
	WJEF54	Ps	E	Y	M	R	SP	-	+	+	+	+	-	-	+	-	+	-	-	-
	WJEF55	Pr	E	Y	M	CB	S	-	+	+	+	+	-	+	+	+	+	+	-	-
	WJEF56	Se	I	Y	M	CB	S	-	+	+	+	+	-	+	+	-	-	+	-	+
	WJEF57	Rh	I	W	V	R	CR	-	+	+	+	+	-	+	+	-	-	+	-	+
	WJEF59	B	I	Y	M	CB	S	+	+	+	+	+	-	+	+	-	-	+	-	-
	WJEF61	R	E	OW	V	CB	S	-	+	+	+	+	-	-	-	-	-	+	-	-
	WJEF62	Ps	E	W	V	R	CR	-	+	+	+	+	-	+	+	-	+	-	-	-
	WJEF63	Rh	E	OW	V	CB	S	-	+	+	+	+	-	+	+	-	-	+	-	+
	WJEF64	Rh	E	Y	M	CB	S	-	+	+	+	+	-	+	+	-	-	+	-	-

Tested positive utilized as substrate; tested negative not utilized substrate. Colony morphology: margin E: entire / I: irregular; color, Y: yellow / W: white / OW: off-white; texture, D: Dry / V: viscid /M: mucoid. Morphology: shapes; R: rod / C: cocci / CB: coccobacilli; arrangement, SP: single pairs / CR: chain rods / S: solitaire. B: Bacillus, Ps: Pseudomonas, Se: Serratia, Str: Streptococcus, Bu: Burkholderia, Rh: Rhizobium, Cl: Clostridium, St: Staphylococcus, Pr: Proteus.

**Table 9:** Genera diversity indices of isolates based on morphological and biochemical characteristics.

	Tafrant	Ait Yaakoub	Dayt Al Amira	Ait Saleh
Taxa_S	5	6	5	6
Individuals	6	9	14	22
Dominance_D	0,2222	0,2099	0,2347	0,2066
Simpson_1-D	0,7778	0,7901	0,7653	0,7934
Shannon_H	1,561	1,677	1,512	1,68
Evenness_e^H/S	0,9524	0,8916	0,9076	0,8947
Brillouin	0,981	1,146	1,168	1,372
Equitability_J	0,9697	0,9359	0,9397	0,9379

#### Relationship between the potential and diversity of PSBs and environmental conditions of the sampling sites

**Fig 7:**
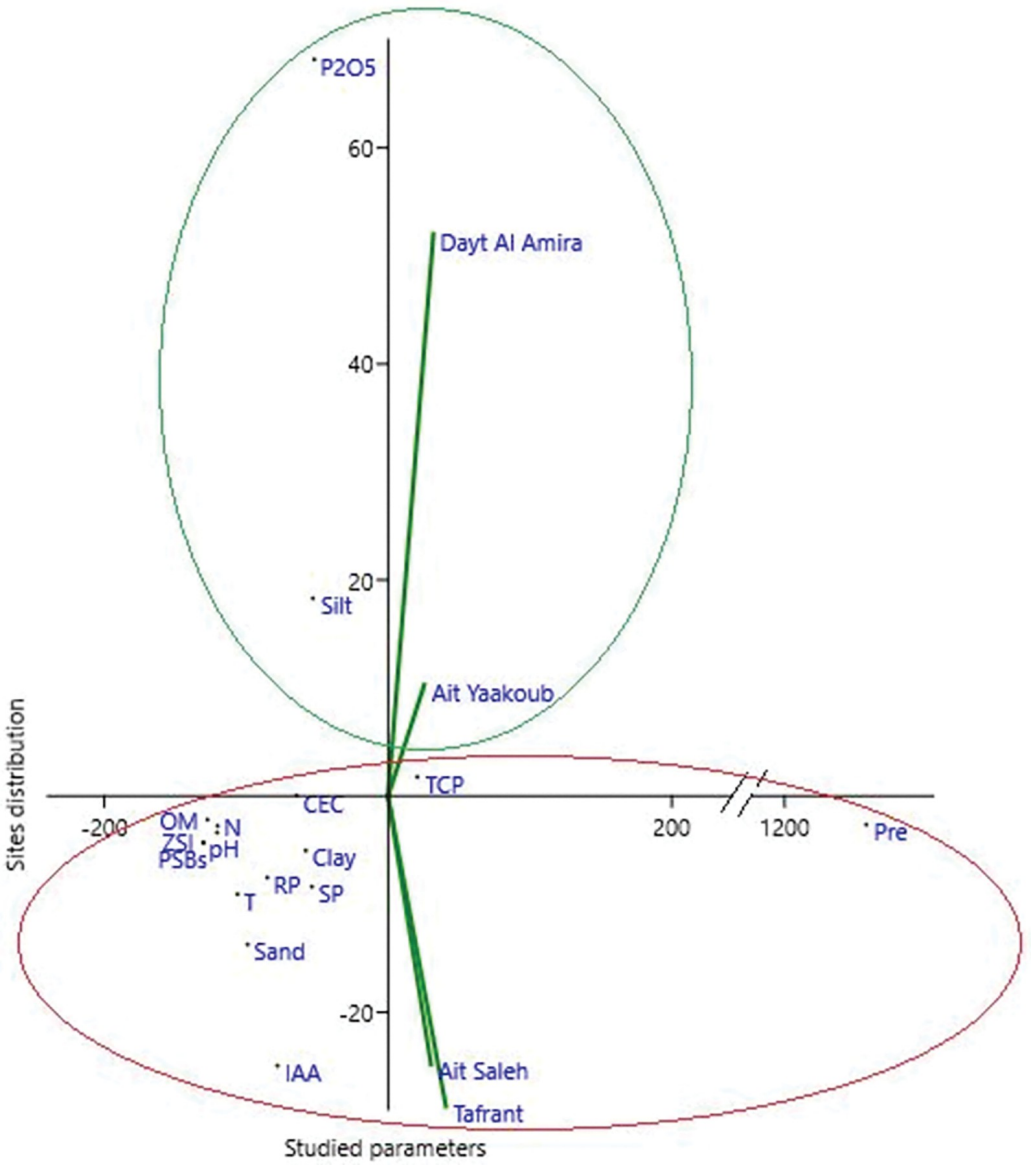
Sites sample distribution using principal component analysis of the various parameters studied. P_2_O_5_: phosphorus pentoxide, TCP: [C] concentration of solubilized tricalcium phosphate, RP [C]: concentration of solubilized rock phosphate, Pre: precipitation, T: temperature, CEC: Cation exchange capacity, OM: organic matter, N: nitrogen mineral, ZSI: zinc solubilization index, SP: siderophores production, IAA [C]: Auxins produced concentration.

The association between the PSBs load, PSBs potential, soil properties, and environmental variables was studied by a dimensional analysis (Principal component analysis (PCA)) (Fig 7) to identify how these variables are correlated with the sampling sites. According to the PCA analysis, the first synthetic component (axis x) corresponds to the studied parameters, while the second synthetic component (axis y) corresponds to the site’s distribution. The studied parameters (environmental condition, soil properties, PSBs load, and PSBs potential) explained 98.5% of the total variation in the distribution of four studied sites. Fig 7 shows that Dayt Al Amira and Ait Yaakoub sites are heavily clustered with the silt and soil P_2_O_5_ concentration and they are in the opposite direction of all studied parameters, where a negative correlation is found. While Tafrant and Ait Saleh sites are positively correlated with all studied parameters except silt and soil P_2_O_5_ concentration. From our results, we can note that the studied sites are subdivided into two groups, in which the soil P_2_O_5_ content has no impact on microbial load or their PGP potential. Whereas, sand, clay, nitrogen mineral, organic matter, pH, cation exchange capacity, and temperature, on the other hand, have a negative effect on PSBs load also their PGP capacity.

The correlation between the ability of isolates to solubilize phosphate on NBRIP medium and selected environmental conditions prevailing at the locations from which soils were sourced was investigated. Based on morphological diversity, Ait Saleh had the highest number of individuals (22 individuals), while Tafrant had the lowest (6 individuals) (Table 9). According to Shannon *H’s* diversity and Dominance *D*, the variation among the four studied sites was somewhat important and close. Tafrant had the highest diversity evenness, while Ait Yaakoub had the lowest.

**Fig 8:**
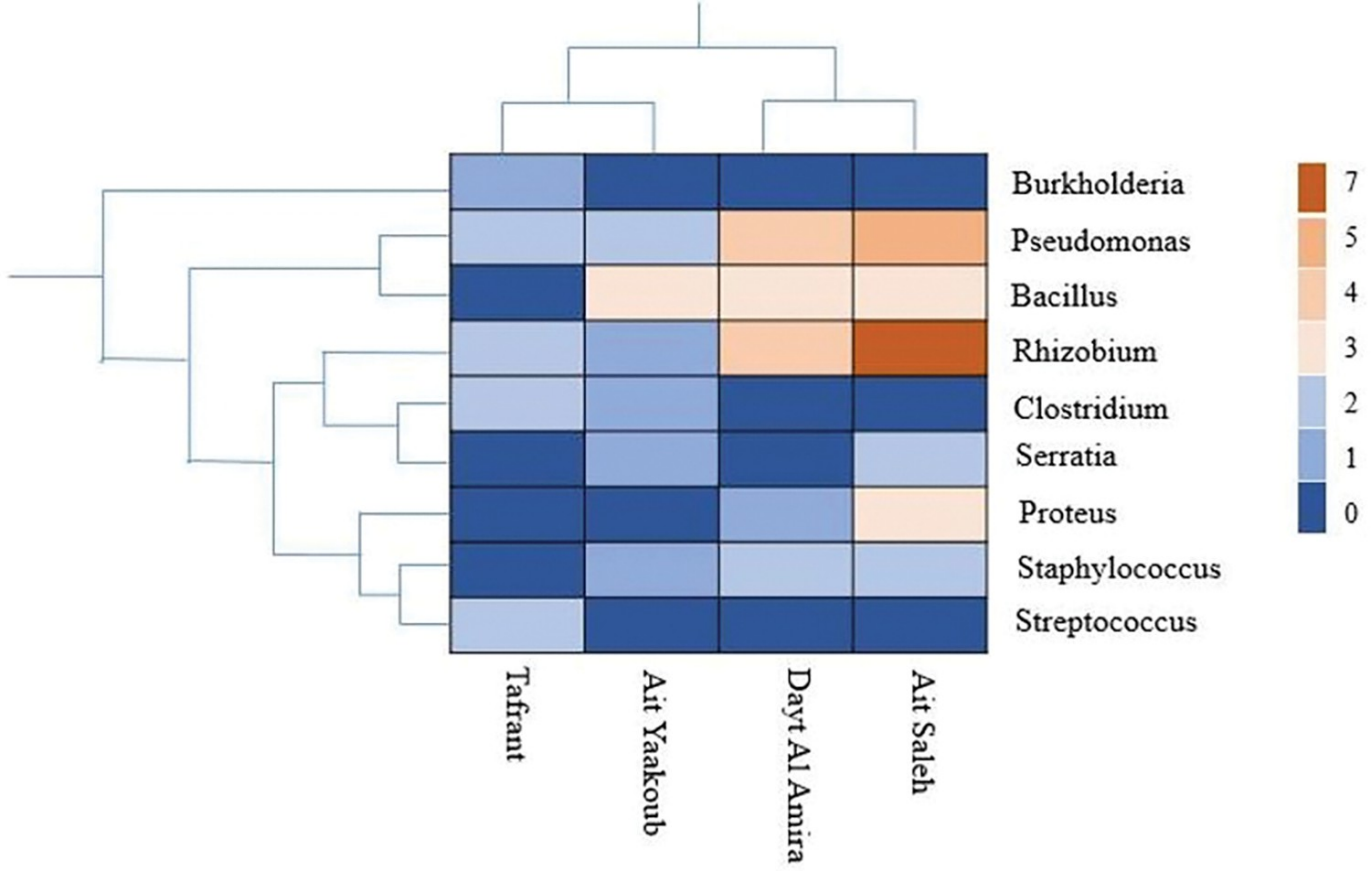
Hierarchically clustered heat map of the bacterial distribution of different communities from the studied rhizosphere at the genus level. A row represents the abundance of each bacterial genus from the identified groups, and a column stands for the studied site samples. The abundance for each bacterial genus was depicted by the colour intensity with the legend indicated at the right of the figure.

A hierarchical heat map of the bacterial distribution at the different studied sites, classified by genus, is presented in Fig 8. The heat map graph illustrated the distribution of each bacterial genus in each site and indirectly in each climate stage. In the present study, the pseudomonas genera were mostly recorded in all sites and the highest and lowest abundance of pseudomonas was found in Ait Saleh and Tafrant, respectively. Rhizobium genera were present in all sites and the maximum abundance was found in the Ait Saleh site. In addition, Bacillus was common in all studied sites except for Tafrant. Rhizobium genera were present in all sites and the maximum abundance was found in the Ait Saleh site. However, Streptococcus and Bulkholderia have been signalled just in the Tafrant site. Among the sites, the highest bacterial diversity (6 genera) was identified in Ait Yaakoub and Ait Saleh.

## Discussion

Soil microbial communities are affected by several factors including soil conditions and environmental changes. These extreme events have serious repercussions on the structure of microbial communities, including the increase in the relative abundance of rare species and the emergence of pathogens capable of infecting plants. The present study focused on the effect of climate and soil conditions on the abundance, potential, and diversity of PSBs from the legumes rhizosphere located at different bioclimatic stages in four regions of Morocco. In addition, to assess the potential impact of these factors on the capacity of PSBs, we tested the P solubilization efficiency of isolates, evaluated their plant’s growth trait potential, and assessed the impact of environmental conditions at each study site on the PSB strains. The selection of an efficient PSB is crucial, as it practically increases P in the plant rhizosphere [2]. Phosphate solubilization capacity is considered a key factor in isolating highly efficient PSBs from agricultural soils [2]. Among One hundred sixty-four (164) PSB strains from different leguminous plants rhizosphere of four sites in the Fez-Meknes region, Fifty-one (51) strains show phenotypic stability of P solubilization after five successive subcultures in NBRIP-RP plates.

To date, only a few studies have focused on the population density of PSBs in different habitats and the factors that influence this. For this reason, we collected information on a place name, geographic location (latitude and longitude), mean annual precipitation (MAP) and mean annual temperature (MAT) of the study sites, soil physicochemical analysis, and the population density of the PSBs (expressed as the number of colony-forming units per kilogram of soil (CFU Kg^-1^)), pH, and total and available P of all samples. As result, all sites differed in both climatic and soil conditions. Soils in the investigated sites can be described as fertile and biologically active, with optimal properties for legume cultivation. Mineralogical compositions of different sites analyzed indicate that Al, Ca, Fe, K, Mg, Na, and P are the most important minerals in the distribution of the various sites. In addition, physicochemical characteristics revealed that the Dayt Al Amira site has the most significant values of the most minerals content with remarkable values of Al, Ca, Mg, Mn, and P. Maximum electrical conductivity, reflecting soil salinity, was recorded in the Ait Yaakoub soil sample of 300.33 (ds.m^-1^) followed with Dayt Al Amira 241.2 (ds.m^-1^), in addition, Dayt Al Amira registers the maximum cation exchange 37.2 (cmol^+^.kg^-1^). We focused on these geographic, climatic, and environmental parameters, as they are potentially important factors influencing the population density of PSBs in the environment [17, 38]. The population density of P-solubilizing microorganisms (PSMs) in environmental samples and its determinants is critical to understanding not only their population ecology but also their roles in regulating the biogeochemical cycling of P and mediating the plant uptake of this element [17]. As stated by Alori et al. (2017) [14], factors influencing microbial P-solubilization abundance include vegetation extent, climatic zone, soil microorganisms interactions, ecological conditions, kinds of soil, plant types, agronomic practices, and soil physical-chemical properties such as organic matter and soil pH. Kavamura et al. (2021) [39] report that edaphic conditions such as differences in soil physical and chemical properties drive microbiome assembly. Soil texture has also been shown to be important in structuring microbial communities as mentioned by Li et al. (2021) [17]. Microbial P-solubilization capacity is mainly regulated by N:P stoichiometry, it is concluded that long-term N inputs decreased microbial P-solubilizing and mineralizing capacity while P inputs favoured microbial immobilization via altering the microbial functional profiles [40]. According to Lai et al. (2022) [41], toxic metal pollution produces serious stress on the reproduction and growth of microorganisms in the soil, and the results of microbial abundance showed that the total microbial biomass in toxic metal-contaminated soil was 42.86%, 53.33%, and 44.93% lower than those in non-contaminated soil. Naher et al. (2021) [42] find that a higher number of total beneficial bacteria is associated with the highest score of soil quality index, with high OM, moderate pH value, and higher concentration of nutrients. Also, Duarah et al. (2011) [43] reported that NPK Fertilization treatments showed a negative effect on the PSBs population, on the other hand, Jana et al. (2001) [44] noted that the abundance of PSBs is closely related to C-N and N-P application. Likewise, He and Wan, (2022) [45] reported that N fertilization treatment enriches culturable tricalcium PSB, phytate-degrading bacteria, and gcd and bpp abundances genes which were more abundant in silt + clay, in addition, P fractions were responsible for PSBs number and P-cycling-related gene abundance. In addition, soil rich in organic matter will favour microbial growth and therefore favour PSMs abundance [14]. Soils at the Ait Saleh site contained a higher content of organic matter, nitrogen, and P available than the other soils site, which showed the highest PSBs load of 3.16x10^4^ (Cells.Kg^-1^ of soil), likely resulting from primary soil properties related to their different pedogenesis such as texture and nutrient abundance. However, research by Hoeber et al. (2020) [46] showed that climatic factors may significantly determine the rate of decomposition of leaf litter which can also be an important source of soil organic matter [1]. The significant positive relationships between soil PSB population densities and available P, NO_3_-N, and dissolved organic carbon in the soil suggest functional couplings between soil PSBs and microbes responsible for soil nitrification and organic matter degradation. In terms of soil pH, it was close to neutral or slightly basic (pH = 8.23 on average), and the soil was moderately calcareous, which probably does not favour the bioavailability of P. Phosphate-solubilizing activities of PSBs are negatively affected by many factors such as temperature, pH, salinity, and dissolved oxygen [7]. Soil pH is classically considered to be the major variable of soil chemistry due to its profound impact on countless chemical reactions [47]. Changes in chemical properties of rhizosphere soil, such as pH and nutrient availability can impact bacterial communities because soil pH is the main driver of microbial community structure including archaeal, bacterial, and fungal members [39]. Soil pH values between 5.5 and 7.5 are the best for P availability, as they avoid P being fixed by aluminium, iron, or calcium, and therefore usable by plants [14].

Besides soil properties, several abiotic factors can affect microbial communities [39]. Abiotic stresses, including warm- or low-temperature environments, adversely affect the structure, composition, and physiological activities of soil microbes. Indeed, temperature is the most vital environmental variable which adversely affects the composition, diversity, community structure, and microbial biomass, and decreases the soil nutrient pool [7]. PSMs tend to be present at higher population densities in hot and humid regions than in dry and cold regions [17]. Except that this was not the case in our study where we found the lowest load of PSBs in the Tafrant site. In this paper, we noted that PSBs abundance was significantly different between the four sites and this was likely a result of differences in the environmental conditions; temperature, humidity, and precipitation. Phosphate solubilizing bacteria surviving at mesophilic, thermophilic, or psychrophilic temperatures release plant-available P from both inorganic and organic P stores. Therefore, they may serve as sustainable and inexpensive alternatives to high-cost and environmentally hazardous chemical P fertilizers [7]. Therefore, the effort is directed to develop a pool of P-solubilizing soil bacteria that grow in the root region and are customized according to the crops and environmental conditions thus releasing plant-accessible P. Likewise, water is one of the most limiting factors for plant development and agricultural losses. Water affects microbial dynamics in three fundamental ways: as a resource, as a solvent, and as a transport medium [48]. The improved soil water and heat status can stimulate soil microorganisms and soil enzymatic activity, influencing the long-term fertility of the soil [49]. The increased water availability may favour the survival of microbes during dry periods by providing a better habitat, leading to enhanced microbial activities, respiration, and growth [50]. Kavamura et al. (2021) [39] investigated the influence of soil water stress history, wheat genotypes with differences in their drought tolerance, and short-term decrease in soil water content on microbial communities of wheat. It was found that the water regime was the main driver of bacterial and fungal community structure in the rhizosphere and root samples of wheat. According to our findings, the Tafrant site has the maximum precipitation but is accompanied by a maximum annual temperature, which directly affects its climatic position in the Emberger diagram. The analysis of this climagram reveals that the station of Tafrant is the only site subjected to the humid bioclimatic stage but warm winter with Q_2_ being approximately 172.57. On the other hand, the Ait Yaakoub region is placed at the limit of Sub-humid and Semi-arid climate with also warm winter and Q_2_ close to 101.23, whereas the two other stations Dayt Al Amira and Ait Saleh classified as Sub-humid climate in soft winter, with Q_2_ close to 126.89 and 126.04, respectively. This explains why the latter two regions have a high abundance of PSBs. Instead, Kałużewicz et al. (2018) [51] noted that lack of water strongly influences the ability of crops to exchange gases, causing stomatal closure [52]. Rising temperatures could make some large agricultural areas unsuitable for cultivation over time [53], and this could be due to increased evapotranspiration and reduced soil microbial quality. We suggest that evapotranspiration is the most important factor shaping microbial diversity. This interference impairs the photosynthetic activity and evapotranspiration of plants, thus affecting plants’ biomass production [54] and microbial biomass diversity. Del Buono (2021) [52] showed that particular CO_2_ enrichment fertilization in the open air can stimulate daily C uptake, plant growth, above-ground biomass production, microbial communities, and soil C, by reducing evapotranspiration for stomatal closure. Similarly, reductions in evapotranspiration cause impairment in the plant’s capacity to uptake nutrients, and indirectly to a lack of sufficient microbial population resources, which explains the high PSBs load in the Ait Saleh site, given the favourable conditions. These results provide further evidence of the functional coupling between PSBs and their geographical location, whereby edaphic and climatic conditions significantly affect their occurrence.

The solubility index on TCP-NBRIP medium was in agreement with previous studies conducted on different plants’ rhizosphere, where the PSBs solubility index ranges from 2 to 3.84 [55–57]. The PSB-isolated strains (from the four sites) used in this work were able to increase P availability in an NBRIP liquid medium containing either RP as the only P source or TCP. Our results support the idea that PSBs are characterized by their ability to easily and efficiently solubilize inorganic P [58–60]. Soluble P concentration in the medium containing RP was between 18.69 mg.L^-1^ and 40.43 mg.L^-1^, while the medium containing TCP was between 71.71 mg.L^-1^ and 94.54 mg.L^-1^. We noted that strains from the Ait Saleh site contain the highest value of solubilization either with TCP or RP. These results are in agreement with other studies done by Elhaissoufi et al. (2020) [61], who report that PSBs can solubilize insoluble inorganic P compounds, such as TCP and RP. Phosphate solubilization is accompanied by a pH medium decrease when compared to control, both in media containing RP and TCP. Furthermore, we reported that as pH values decrease, the P concentration increases. This is noted by Batool & Iqbal, (2019) [62] who indicate a strong correlation between P solubilization and pH decrease. The acidic (lowering of pH) environment of the medium suggests the release of organic acids that occurs on the outer face of the cytoplasmic membrane via the direct oxidation pathway [63]. Many reports have established a positive correlation between pH drop and soluble P concentration in the liquid culture medium [7]. In addition, we noted that strains isolated from Ait Saleh and Dayt Al Amira sites (classified as Sub-humid climates in soft winter) were the most efficient PSBs regarding their P solubilizing capacity, which also explains the PSBs load in these regions taking into account the availability of P and the pH value. We hypothesize that the available P in soil may influence PSBs communities in the rhizosphere to some extent. Li et al. (2021) [17] reveal that the population density of PSMs in continental and global environmental samples is regulated by total P rather than pH. Bodenhausen et al. (2019) [64] found marked root microbiota changes in Arabidopsis between low-P and high P-fertilized treatments, while Huang et al. (2016) [65] indicated that soil microbes were insensitive to an elevated P availability in a subalpine spruce plantation. However, our analysis suggests that climate (temperature and precipitation) interact with soil physicochemical conditions to affect soil P availability and indirectly PSBs occurrence and diversity. Although P-solubilization ability is common in the genus *Bacillus* and *Pseudomonas*, various species and strains differ in their capacity to mobilize P due to differences in the secretion of organic acids, phosphatases, and phytase or other P-solubilizing mechanisms [66]. Several studies use *Bacillus* [67] and *Pseudomonas* [68] species as bioinoculants that improve P availability for various plants. In our study, we found that *Pseudomonas* is the dominant genus of PSBs in the group of isolated strains, followed by the *Bacillus* genus. Less frequent isolates were of the *Streptococcus* and *Burkholderia* genera. In contrast, the most efficient P-solubilizing strain was the *Rhizobium* genus. This effect might be based on the varying site-specific environmental conditions and their interactions with the genotypes. Zhang et al. (2019) [69] mentioned that soil pH, soil OM, fluorescein diacetate hydrolase activity, and soil bioavailable Cd were the dominant environmental factors that significantly influenced the microbial relative abundance and explained most of the variance in the species-environment correlations. Zhu et al. (2022) [70] pointed out that organic matter content, oxygen content, pH, and total phosphorus were identified as important environmental factors determining the functional diversity of bacterial communities and survival, while pH influences species evolution.

Among the PGP traits, auxins are powerful growth hormones produced naturally by plants. They are found at the tips of shoots and roots and support cell division as well as stem and root growth. They can also influence plant orientation by directing cell division to one side of the plant in response to sunlight and gravity [71]. Indole-3-acetic acid (IAA) is the most common naturally occurring plant hormone of the auxin class; thus, in our next study, we anticipate verifying and quantifying specifically the IAA production using High-performance liquid chromatography HPLC to provide additional details on the potency of PSBs concerning their source samples. The results show the capacities of PSBs to solubilize zinc, production of auxins and siderophores, and ACCD activity, which were positively correlated with PSB’s load and site location from which the strains were isolated. However, this result alone did not demonstrate whether the ability of isolates to enhance PGP traits would assist the bacteria themselves to withstand the stress conditions. Alemneh et al. (2022) [9] suggest that water stress and low soil fertility with higher levels of Cu, Mn, and Zn at the source site can result in the selection of rhizobacteria with higher IAA production potential. Likewise, Malhotra & Srivastava, (2009) [72] noted that nitrogen starvation that suppresses cell viability can increase IAA production by *Azospirillum brasilense SM* in vitro. Zinc solubilization results show a wide variation by isolates recovered from rhizosphere soil samples collected from different agricultural and soil management practices on nutrient agar medium. The isolates exhibited a significant variation in zinc solubilization. Maximum zinc solubilization was observed for zinc phosphate followed by zinc oxide. These findings are in accordance with Ramesh et al. (2014) [73] who reported maximum solubilization for zinc phosphate but contrary to Yasmin et al. (2021) [28] who found maximum solubilization for ZnO. An increase in available zinc in the medium from the Zinc-P compound was also accompanied by a reduction in pH that could be correlated with organic acids production such as gluconic acid, in which, acidification appears to be the main strategy followed by zinc-solubilising strains [28]. Organic acids or exudative chelating agents can be said to be acting as agents causing zinc solubilization and an important phenomenon in metal solubilization. Carbon and N sources play a modulating role in the synthesis of secondary metabolites and the release of Zn from insoluble Zinc-P compounds [74]. Although some contribution may also come from the excretion of other metabolites, siderophores and CO_2_ from respiration, the importance of all these processes is variable and dependent on the organisms and growth conditions [73]. The ability of bacteria to solubilize all four inorganic zinc compounds might be interesting as zinc is known to occur in soils in discrete chemical forms varying in their solubility and availability to crop plants [73]. Bacteria from the Ait Saleh region dominate this assay and represent 75% of the total zinc-solubilizing bacteria. This can be attributed to location specificity in terms of varying organic carbon content, and species and strains variability [75]. In addition, the production of siderophores is a PGP feature that may play an important role in biocontrol and mobilizing phosphorus by mineral chelation [76]. Among the role of siderophores, they can solubilize and sequester iron from the soil, but they may also play an indirect role in plant growth promotion [77], which explain the high Fe values in the Dayt Al Amira and Ait Saleh regions, whose PSBs load is the highest. Among the more current methodology, the use of natural plant biostimulants like PSB strains has been proposed to improve plant resistance to abiotic environmental stresses. The advantage of using these substances is due to their effectiveness in improving crop productivity and quality [52]. Thus, adapted bacteria can be used as biofertilizers, biocontrol agents, and bioremediation for augmenting the growth and yield of crops grown in different ecological areas.

## Conclusion

This research work provides valuable insights into the PSB’s density in different habitats. The main objective of this study was to evaluate the effect of climate and soil conditions on the abundance and diversity of PSBs in four varied regions in Morocco. In addition, we tested the P solubilization efficiency and the PGP traits of isolates to assess the potential impact of the cited factors on the competencies of PSBs. The density of the PSBs population was significantly different between the four sites due to varied environmental conditions: temperature, humidity, and precipitation of the selected sites. The abundance of PSBs and PGP traits were positively correlated as the Ait Saleh site, the most PSBs load, show 70% and 69% of auxins and siderophores production, respectively. Based on the mentioned results, the climate appears to exert the most selective pressure on PSBs phenotypes such as P solubilizing competence. Also, we noted that climate influences the structure of PSBs communities in the studied sites. In general, this study makes a significant contribution to the sustainable agriculture field by conducting a thorough investigation of PSBs’ performance associated with the environmental conditions of their soil source. We suggest extending the field of work and investigating different regions that are more likely to contain efficient PSBs with traits and ecological mechanisms that can improve agricultural production as well as directing research toward appropriate pathways.

## Supporting information

S1 Table All PSBs strain’s P solubilization. Values for both RP and TCP liquid NBRIP medium.
(PDF)
