# Supplementary material for: Phosphate solubilizing bacteria from soils with varying environmental conditions: Occurrence and function
Source: PLoS One. 2023 Dec 8;18(12):e0289127. doi: 10.1371/journal.pone.0289127 (PMC10707511; doi:10.1371/journal.pone.0289127)
Supplement: S1 Table — Values for both RP and TCP liquid NBRIP medium. (PDF) [file pone.0289127.s001.pdf]

All PSBs strain's P solubilization values for both RP and TCP liquid NBRIP medium:

Tafrant

Strains	RP pH	RP [C]	TCP pH	TCP [C]
WJEF2	4,3825	22,5985257	4,875	65,01284247
WJEF3	4,4525	19,5576978	5,055	66,875
WJEF4	4,6675	12,3702864	4,84	78,51883562
WJEF4'	4,4275	20,4366317	4,76	64,30650685
WJEF5	4,2925	22,6552311	4,91	69,43379791
WJEF8	4,78	14,518004	4,84	86,13675958
Means	4,50041667	18,6893961	4,88	71,71395707

Ait Yaakoub

Strains	RP pH	RP [C]	TCP pH	TCP [C]
WJEF9	4,8025	13,518571	4,54	89,9259582
WJEF10	5,195	12,7714235	4,765	79,8886986
WJEF11	4,205	31,4675348	4,86	78,2833904
WJEF12	4,645	26,4173852	4,655	51,1857877
WJEF13	4,92	15,6660215	4,955	62,6797945
WJEF15	4,275	72,6420904	4,69	75,6292808
WJEF16	4,555	13,0436632	4,445	86,3545296
WJEF17	4,46	24,9832835	4,965	87,1875
WJEF18	4,93	16,6426183	4,66	68,5191638
Means	4,665277778	25,2391768	4,726111111	75,5171226

Dayt Al Amira

Strains	RP pH	RP [C]	TCP pH	TCP [C]
WJEF21	4,3625	23,5554295	4,73	77,2714808
WJEF22	4,4375	16,9988659	4,745	60,0868373
WJEF24	4,75	14,2557414	4,62	66,1654479
WJEF25	5,055	16,7393982	4,645	82,1846435
WJEF26	4,675	53,7480204	4,67	78,6654479
WJEF28	4,805	14,4967394	5,065	84,28484
WJEF29	4,8025	12,6892543	4,785	74,5584892
WJEF30	4,735	15,0779699	4,94	78,4271726
WJEF31	4,805	13,6072497	4,96	71,1269453
WJEF32	4,505	24,9744853	4,52	89,3993705
WJEF33	4,37	27,6139363	4,48	99,1257213
WJEF34	4,35	29,8574696	4,49	83,9351285
WJEF36	5,26	18,7663927	5,26	68,7663927
WJEF37	4,91	11,434647	4,85	75,8043364

Means	4,70160714	20,9868286	4,76857143	77,8430181

Ait Saleh

Strains	RP pH	RP [C]	TCP pH	TCP [C]
WJEF38	4,75	77,2611297	4,23	120,195838
WJEF39	4,5975	15,4111143	4,535	85,4869732
WJEF40	4,695	16,5634348	4,605	77,8588914
WJEF41	4,6325	73,807848	4,22	123,102815
WJEF43	5,2	11,8386731	4,33	129,725476
WJEF44	4,635	14,5134612	4,885	86,0115405
WJEF45	4,52	74,2917473	4,225	88,7603378
WJEF46	4,515	74,5776878	4,195	100,813831
WJEF49	4,755	20,9009326	4,255	112,999296
WJEF50	5,13	10,8534225	4,905	59,9243357
WJEF51	4,67	80,7364068	4,145	99,5161007
WJEF52	4,5675	14,4329458	4,175	102,287524
WJEF53	4,465	14,8298838	4,175	101,517684
WJEF54	4,71	17,1881049	4,875	56,2291043
WJEF55	5,075	11,8564139	4,835	56,5810311
WJEF56	4,575	47,8312511	4,775	75,6070737
WJEF57	4,725	20,14429	4,765	64,3454162
WJEF59	4,555	78,4488826	4,345	127,494281
WJEF61	4,66	79,4606722	4,3	147,620095
WJEF62	4,53	24,7721274	4,41	80,8199894
WJEF63	4,78	76,5792715	4,2	114,934894
WJEF64	3,92	33,1743797	4,871	67,983562

All PSBs strain's P solubilization index on solid NBRIP-TCP medium:

Tafrant		Ait Yaakoub			
Strains	PSI	Strains	PSI	Strains	PSI
WJEF2	2.29	WJEF9	1.90	WJEF16	2.22
WJEF3	2.47	WJEF10	2.15	WJEF17	2.29
WJEF4	2.31	WJEF11	2.54	WJEF18	2.36
WJEF4'	2.56	WJEF12	2.38		
WJEF5	2.50	WJEF13	2.07		
WJEF8	3.84	WJEF15	2.46		
Dayt Al Amira					
WJEF21	2.09	WJEF29	1.40	WJEF36	2.31
WJEF22	2.75	WJEF30	2.57	WJEF37	2.39
WJEF24	2.85	WJEF31	2.15		
WJEF25	2.90	WJEF32	2.33		
WJEF26	2.67	WJEF33	2.50		
WJEF28	2.67	WJEF34	2.33		
Ait Saleh					
WJEF38	2.30	WJEF49	2.50	WJEF56	2.33
WJEF39	2.50	WJEF50	2.33	WJEF57	2.30
WJEF40	2.78	WJEF51	2.33	WJEF59	2.30
WJEF41	2.27	WJEF52	2.40	WJEF61	2.14
WJEF43	2.20	WJEF53	2.33	WJEF62	2.64
WJEF44	2.09	WJEF54	2.22	WJEF63	2.33
WJEF45	2.30	WJEF55	2.11	WJEF64	2.17
WJEF46	2.27				

Table. The average PSI in NBRIP-TCP solid medium of all studied strains of each sampling site.

**Sites**	**Tafrant**	**Ait Yaakoub**	**Dayt Al Amira**	**Ait Saleh**
PSI	2.66	2.26	2.42	2.33
